# *Salvia elegans* Vahl Counteracting Metabolic Syndrome and Depression in Mice on a High-Fat Diet

**DOI:** 10.3390/molecules29174070

**Published:** 2024-08-28

**Authors:** Gabriela Belen Martínez-Hernández, Enrique Jiménez-Ferrer, Manases González-Cortazar, Zamilpa Alejandro, Nayeli Monterrosas-Brisson, Maribel Herrera-Ruiz

**Affiliations:** 1 Centro de Investigación Biomédica del Sur, Instituto Mexicano del Seguro Social (IMSS), Argentina 1, Xochitepec 62790, Mexico; gabmh89@gmail.com (G.B.M.-H.); enriqueferrer_mx@yahoo.com (E.J.-F.); gmanases@hotmail.com (M.G.-C.); azamilpa_2000@yahoo.com.mx (Z.A.); 2 Facultad de Ciencias Biológicas, Universidad Autónoma del Estado de Morelos (UAEM), Cuernavaca 62209, Mexico; nayeli.monterrosas@uaem.mx

**Keywords:** dyslipidemia, obesity, hypertension, central and systemic inflammation, inflammatory cytokines, serum corticosterone, sterols, phenolic compounds

## Abstract

*Salvia elegans* Vahl is a plant commonly used in Mexico as a remedy for nervous disorders, inflammatory diseases, and “ringing in the ears”; the latter can be associated with arteriosclerotic conditions and arterial hypertension. Therefore, based on medicinal use, this work aimed to evaluate the hydroalcoholic extract (SeHA, 100 mg/kg) of this plant and two fractions, ethyl acetate (SeFAc, 50 mg/kg), and obtained from SeFAc fractionation denominated SeF3 (10 mg/kg), on several alterations derived from metabolic syndrome (MetS) derived from the ingestion of a high-calorie diet (high-fat diet), in ICR (Institute of Cancer Research) mice, leading to chronic inflammation that results in neurological damage such as depression. Therefore, several MetS-related parameters, such as forced swim tests, hypertension, serum corticosterone levels, glucose, triglycerides, cholesterol, adiposity index, and insulin resistance, will be evaluated. Additionally, tumor necrosis factor (TNF)-α, interleukin (IL)-1β, IL-6, and IL-10 levels were measured in kidneys, fat tissue, brains, and spleens. It was proven that all those *S. elegans*-derived treatments reversed the damage, showing antidepressant, antihypertensive, antihyperglycemic, and antidyslipidemic effects and decreased adiposity, insulin resistance, and serum corticosterone. They induced a modulatory response by modifying the levels of TNF-α, IL-1β, IL-6, and IL-10 in different organs. High-performance liquid chromatography (HPLC) analysis of the acetate of ethyl fraction from *S. elegans* (SeFAc) fraction revealed the presence of rosmarinic and caffeic acids as well as flavonoids, while the fraction from SeFAc called SeF3 Was identified by gas mass as methyl glucose, glycerol, and known sterols, among others. Thus, it was concluded that *S. elegans* protects against the harmful effects of MetS.

## 1. Introduction

Depression is a global public health problem affecting more than 300 million people [1]. Incidence and severity are increasing worldwide, and there is a direct correlation between this disorder and metabolic syndrome (MetS) [2], which is a constellation of conditions (hyperglycemia, hypertension, waistline body fat, high cholesterol, and triglyceride levels), that may increase the risk of stroke, heart disease, and diabetes. It is crucial to emphasize the impact of obesity and its determinant role in MetS since, in 2016, more than 1900 million adults worldwide were found to be overweight, and 650 million were obese. Global prevalence of obesity increased almost three-fold between 1975 and 2016 [3].

Overactivation of the hypothalamic pituitary adrenal (HPA) axis elevates cortisol (corticosterone in rodents). The overactivation of the hypothalamic–pituitary–adrenal (HPA) axis elevates cortisol (corticosterone in rodents). It has been shown that chronic hypercortisolism can alter HPA in patients with depression and MetS, leading to hyperglycemia, dyslipidemia, and hypertension [4,5]. It also affects the innate immune response by causing low-grade systemic inflammation, which increases proinflammatory cytokines, such as tumor necrosis factor-alpha (TNF-α), in obese people [6,7]. Depression is also associated with increased chronic inflammation. Furthermore, in depression, levels of interleukin (IL)-1 and IL-6, activators of the HPA axis, also trigger MetS [8]. Therapeutically, depression is treated with tricyclic antidepressants (Imipramine), serotonin and norepinephrine reuptake inhibitors (SNRI), and selective serotonin reuptake inhibitors (SSRI), among others [9]. MetS is treated with antihypertensive, antidyslipidemic, and antihyperglycemic drugs with the goal of reducing vascular damage.

The use of natural products to treat MetS is increasing [10], and the genus *Salvia* has been evaluated in various animal models of cardiovascular and neurological diseases [11]. In Mexico, “mirto” or “pineapple sage” (*Salvia elegans* Vahl, Lamiaceae) is used additionally as a tranquilizer, sedative, and analgesic for antidizziness and against “buzzing in the ears”, the latter attributed to arteriosclerotic and hypertensive disorders [12]. This plant is used in gastronomy for having aromatic characteristics and a pleasant flavor [13]. *S. elegans* exerts anxiolytic and antidepressant [14,15]; antihypertensive [16], specifically as Angiotensin Converting Enzyme (ACE) inhibitors [16,17]; antioxidant and α-glucosidase inhibitor [18]. Some compounds reported in this species includes α-amirin; β-amirin; 3,4-secoisopimar-4; 7,15-triene-3-oic acid; ursolic acid; oleanolic acid; corosolic acid; maslinicacid; 3′,5-dihydroxy-4′,6,7, trimethoxyflavone (eupatorin); 3′,5-dihydroxy-4′,7,8, trimethoxylavone; uvaol; erythrodiol; caffeic acid; rosmarinic acid; Isosakuranetin-5-*O*-rutinoside, neoeriocitrin; luteolin glucoside and isosakuranetin-5-*O*-glucosyl (10′′′ → 20″) rhamnosyl (10″ → 60′) glucoside [17].

The aim of the present study was to establish the pharmacological effect of *S. elegans* on depression and MetS in a murine model of high-fat food-induced obesity.

## 2. Results

### 2.1. HPLC Chromatographic Profile of the SeHA Extract and the SeFAc Fraction

**Figure 1:**
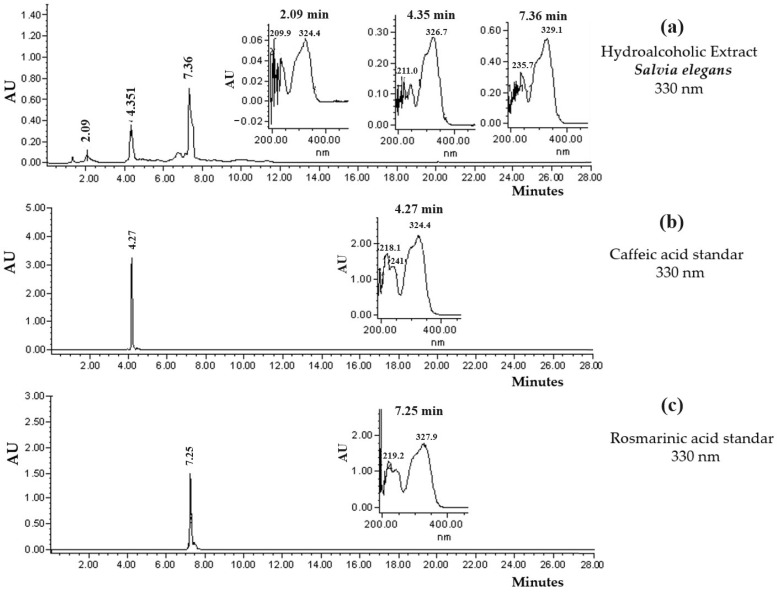
HPLC chromatogram (330 nm) of (**a**) SeHA extract, UV light spectrum of the compound, (**b**) caffeic acid (4.3 min) standard, and (**c**) rosmarinic acid (7.3 min) standard.

The analysis of SeHA with high-performance liquid chromatography (HPLC) allowed us to identify three main compounds. The latter exhibited retention times of 7.3, 4.3, and 2.0 min (Figure 1a). According to their ultraviolet (UV) spectra, as well as to a comparison with the data described in the literature [16], it was found that they were phenolic compounds, known as caffeic acid (4.3 min, Figure 1b), rosmarinic acid (7.3 min, Figure 1c), and an unidentified compound (2.0 min).

**Figure 2:**
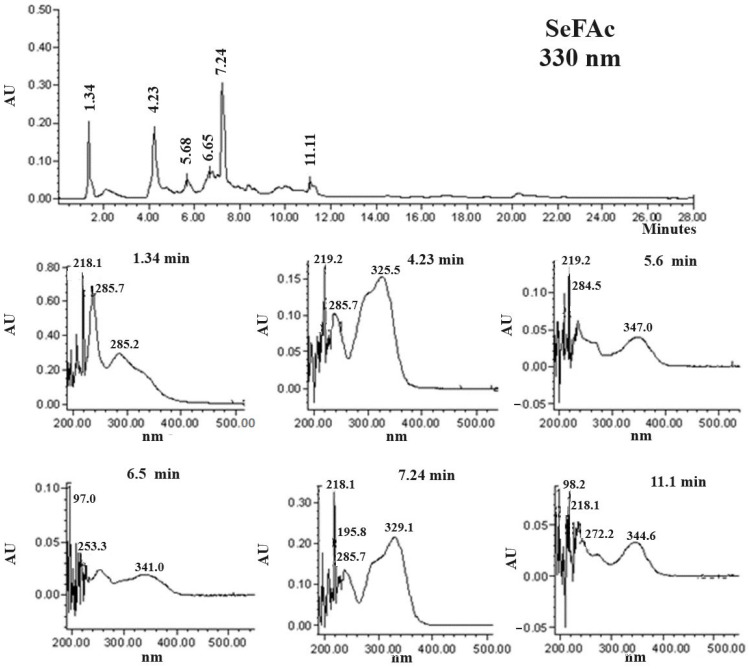
HPLC chromatogram (330 nm) of the acetate of ethyl fraction (SeFAc) and UV light spectrum of each existing compound.

**Figure 3:**
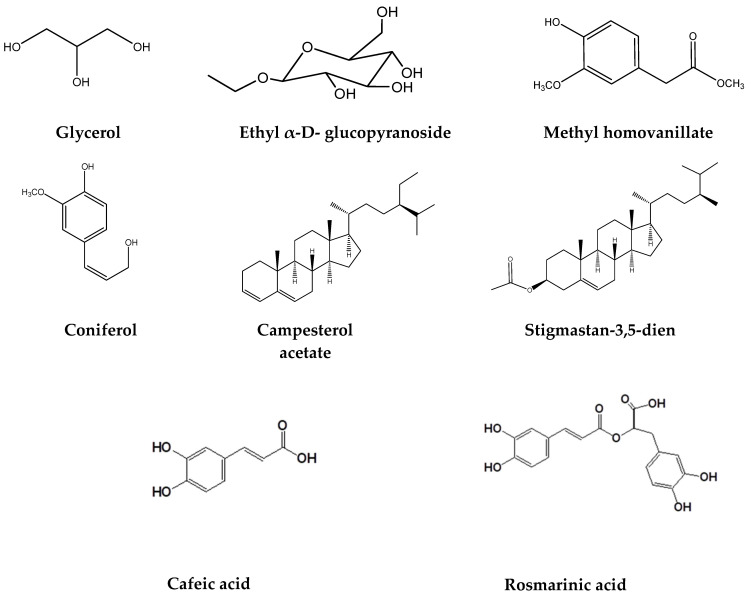
Structures of compounds in SaFAc or SeF3 from *S. elegans*.

SeFAc exhibited six major compounds with retention times of 1.34, 4.2, 5.6, 6.6, 7.2, and 11.1 min (Figure 2). According to their UV light spectra (Figure 2), they matched with phenolic-type compounds such as caffeic (4.2 min) and rosmarinic (7.2 min) acids (Figure 3), as well as unidentified flavonoids.

### 2.2. Chemical Composition of the SeF3 Fraction by GS-MS

The SeF3 fraction was analyzed by gas chromatography coupled to mass spectrometry, and the identified compounds in high concentration were glycerol **1**, α-d-glucopyranoside ethyl, coniferol, campesterol acetate, stigmastane-3,5-diene, and stigmastane-3,5-diene acetate (Table 1, Figure 3). The compounds obtained were identified by fragmentation comparison with those in the National Institute of Standards and Technology (NIST, Library 1.7) library.

### 2.3. Effect of S. elegans in FST and on Serum Corticosterone Concentration

**Figure 4:**
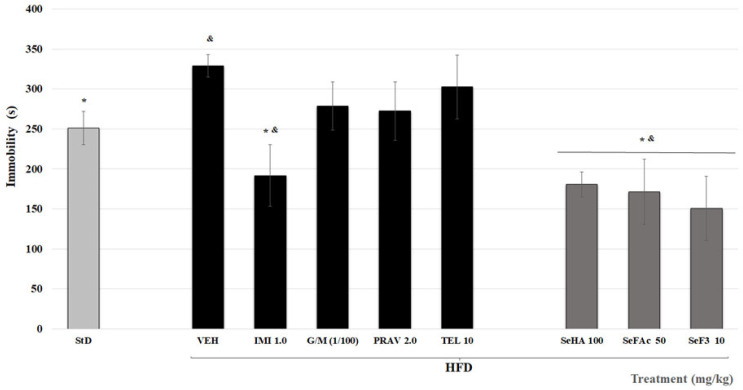
Effect *S. elegans* treatments (SeHA = hydroalcoholic extract; Se-FAc = acetate of ethyl fraction; SeF3 = sub-fraction from SeFAc) and from different drugs (IMI = Imipramine; G/M = Glibenclamide/Metformin; PRAV = Pravastatine; TEL = Telmisartan) on immobility time (A) of mice with a high-fat diet (HFD) exposed to a forced swimming test (FST). ANOVA post-test Bonferroni (* *p* ≤ 0.05, in comparison with the VEH group; (^&^ *p* ≤ 0.05, in comparison with standard diet (StD) group; *n* = 10).

Chronic high-fat diet (HFD) increased immobility time in the forced swimming test (FST); these data were significantly different from StD animals (* *p* < 0.05). Imipramine (IMI)-treated animals exhibited significantly less immobility time compared to the standard diet (StD) and HFD groups (^&^, * *p* < 0.05, Figure 4). There was no such effect for the other animals administered with telmisartan (TEL), glibenclamide plus metformin (G/M), or pravastatin (PRV) (*p* > 0.05). All treatments from *S. elegans* decreased the immobility time compared to the StD and HFD groups (^&^, * *p* < 0.05; Figure 4).

**Figure 5:**
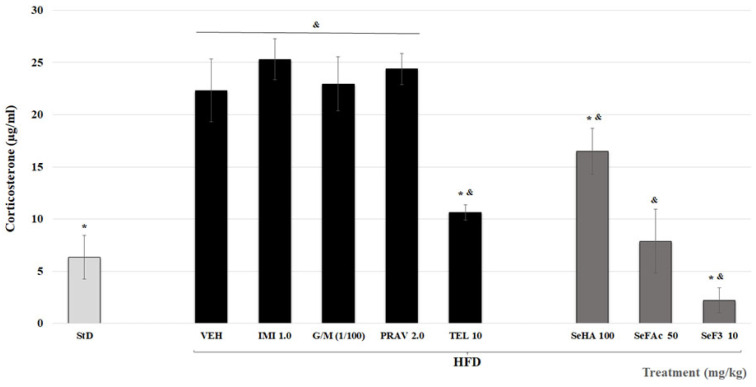
Effect of treatments from *S. elegans* (SeHA = hydroalcoholic extract; SeFAc = acetate of ethyl fraction; SeF3 = sub-fraction from SeFAc) and from different drugs (IMI = Imipramine; G/M = Glibenclamide/Metformin; PRAV = Pravastatin; TEL = Telmisartan) on the serum concentration of corticosterone in mice with a high-fat diet (HFD). ANOVA post-test Bonferroni (* *p* ≤ 0.05, in comparison with the VEH group; (^&^ *p* ≤ 0.05, in comparison with the standard diet (StD) group; *n* = 10).

VEH corticosterone concentration was higher than that of the StD group (* *p* < 0.05). Animals treated with IMI, TEL, or any *S. elegans* treatments showed less concentration of this hormone than VEH (* *p* < 0.05; Figure 5).

### 2.4. Effect of S. elegans on MetS-Associated Parameters in HFD Mice

Weight gain was higher in animals subjected only to HFD (VEH) compared to those subjected to StD (^&^ *p* ≤ 0.01), while IMI, G/M, PRV, TEL, as well as SeFAc, and SeF3 decreased weight compared to HFD (* *p* ≤ 0.01; Table 2), SeHA did not modify such data.

HFD intake increases SBP and DBP at 8 and 12 weeks, compared with Std animals (^&^ *p* ≤ 0.01). IMI and PRV drugs did not inhibit blood pressure rise, whereas G/M avoided elevation of systolic (SBP) and diastolic (DBP) blood pressure until the 12th week. However, TEL, SeHA, SeFAc, and SeF3 reduced SBP and DBP at values similar to those of the standard diet group from week 8 onward, and their values were statistically different compared with those of the VEH group (* *p* ≤ 0.01; Table 2). HFD mice had increased serum glucose, triglycerides, and cholesterol (^&^ *p* ≤ 0.05; Table 2). Administration of G/M, PRAV, and SeF3 reduced glucose levels (* *p* ≤ 0.01; Table 2). Simultaneously, PRV, TEL, SeFAc, and SeF3 inhibited the other variables (* *p* ≤ 0.01), IMI and G/M just avoided the rise in triglyceride levels (* *p* ≤ 0.01), while SeHA stopped cholesterol increase (* *p* ≤ 0.01).

The AUC of insulin resistance was higher in the HFD group than in the StD group (^&^ *p* ≤ 0.01). All treatments, including the reference drugs and those from *S. elegans*, counteracted this effect (* *p* ≤ 0.01; Table 2). The adiposity index was higher in animals with the HFD diet compared to those of the StD diet (^&^ *p* ≤ 0.01); nevertheless, this effect was only modified with PRV and SeF3 (* *p* ≤ 0.01); the other treatments did not change this condition (Table 2).

### 2.5. S. elegans Effects on Different Organ Cytokines in Mice with MetS-Induced HFD

Daily HFD intake in mice modified IL-10 and proinflammatory cytokine levels in all organs analyzed (Table 3). Thus, in the left and right kidneys, adipose tissue, and the brain, TNF-α, IL-1β, and IL-6 proteins increased compared to animals consuming only the StD diet. Whereas in the spleen, these proteins decreased with respect to those of the StD diet (^&^ *p* ≤ 0.05). IL-10 concentration increased in both kidneys and adipose tissue, whereas a decrease was observed in the brain and spleen, compared to healthy mice (StD, ^&^ *p* ≤ 0.05).

#### 2.5.1. Kidneys

##### Left Kidney

While all treatments decreased TNF-α, IL-1β, and IL-6 levels in the left kidney, IMI, G/M induced a significant decrease, and PRAV in IL-10 levels in comparison to the VEH group (* *p* < 0.05), and no change was induced by TEL (*p* > 0.05). In addition, all cytokines also decreased with SeF3, while only IL-1β decreased with SeHA and SeFAc raised IL-6 but decreased the remaining cytokines, as compared to the VEH group data (* *p* ≤ 0.05; Table 3).

##### Right Kidney

Reference drugs, used as controls, promoted an increase in TNF-α levels and lowered IL-6 ones, compared to the VEH group. IL-1β ones, reduced with IMI, G/M, and TEL. Regarding the anti-inflammatory cytokine IL-10, PRAV significantly increased the concentration of this cytokine, while IMI and TEL both reduced it. SeF3 reduced the local concentration of all cytokines. In addition, SeFAc administration increased TNF-α and elicited the reduction of IL-1β and IL-10. These data were significantly different from those of the VEH group. (* *p* ≤ 0.05; Table 3).

#### 2.5.2. Fat Tissue

The results in adipose tissue revealed that IMI and TEL decreased TNF-α, IL-1β, and IL-6 in this tissue while increasing IL-10 (Table 4). Whereas G/M just modulated IL-1β, causing a reduction of this molecule. PRAV lowered TNF-α levels compared to the VEH group (* *p* ≤ 0.05; Table 4).

TNF-α and IL-1β levels decreased in this tissue in the presence of all three *S. elegans* treatments, while IL-6 was lower than in the VEH group just with SeFAc and SeF3. IL-10 diminished in animals that received SeFAc (* *p* ≤ 0.05; Table 4).

#### 2.5.3. Brain

In this organ, a reduction of TNF-α was observed with IMI, PRAV, TEL, and SeHA, but the concentration of this cytokine was not affected by G/M or SeF3. By contrast, this molecule was higher in the group administered with SeFAc (* *p* ≤ 0.05; Table 5). Cytokine IL-1β increased with all synthetics except PRAV, which caused a reduction (* *p* ≤ 0.05; Table 5). Concerning IL-6, all treatments lowered the concentration of this molecule in the brain. Only IMI, TEL, and all fractions increased in their concentration (* *p* ≤ 0.05; Table 5).

#### 2.5.4. Spleen

All treatments, except SeF3, caused an increase in IL-6 and IL-10 concentrations in the spleen. TNF-α and IL-1β decreased with IMI. For the G/M group, both cytokines increased. On the one hand, TEL promoted TNF-α increase; on the other, the decrease of IL-1β. Finally, SeF3 reduced IL-1β (* *p* ≤ 0.05, Table 6).

## 3. Discussion

Stress and inflammation are detrimental consequences of MetS that are linked to obesity and nervous disorders, such as depression. Considering the comorbidity between psychiatric disorders and MetS, we evaluated the effect of *S. elegans* on secondary depression related to high corticosterone levels in obese mice. The results of the present study suggest that SeHA, SeFAc, and SeF3 treatments reduce the damage parameters on the affected organs according to the anxiolytic, antidepressant [14,15,18,19], and antihypertensive [16,17] effects previously described.

Likewise, SeHA, SeFAc, and SeF3 in obese mice decreased immobility time in FST like that of the antidepressant drug IMI. This activity was observed in non-obese (healthy) mice [14,18] and those using the tail suspension test [19]. Depression in obese mice exposed to HFD was secondary to an increase in serum corticosterone concentration compared to StD mice. This parameter decreased when IMI, TEL, SeHA, SeFAc, and SeF3 treatments were administered. FST causes sustained corticosterone to increase in rats [20], which did not decrease with the administration of IMI at 5 mg/kg [21]. Increased fat in rodents’ daily diet is considered a stress factor, which over-reactivates the HPA axis, related to the development of MetS in experimental rodents [22]. The antidepressant effect could be attributed to the phenolic and flavonoid components reported for *S. elegans*, for example, caffeic acid (4 mg/kg), rosmarinic (2 mg/kg) acid [14], and isosakuranetin-5-O-rutinoside (15 mg/kg) [19].

*S. elegans* mitigated MetS alterations in obese mice exposed to HFD, as SeFAc and SeF3 controlled weight gain, which was not the case with SeHA. The effects of antihyperglycemic, antidyslipidemic, antiadiposity, and reduced insulin resistance were also demonstrated for this plant. These activities may be related to the ability to inhibit α-amylase and lipase enzymes previously described in *S. elegans*, which are related to its content of apigenin, rosmarinic acid, luteolin, caffeoyl, caffeic acid, and their derivatives, as well as flavones [23].

SeHA and SeFAc were able to manage the typical hypertensive component of MetS, attributed to the presence of rosmarinic and caffeic acids, isosa-kuranetin-5-*O*-rutinoside, and neoeriocitrin that inhibit angiotensin-converting enzyme (ACE) [16,17]. This evidence could explain the antihypertensive activity observed in obese mice exposed to HFD in the sense of modifying the over-reactivation of the renin-angiotensin-aldosterone system (RAAS).

Rosmarinic acid from *S. elegans* has been shown to counteract MetS by antagonizing the peroxisome proliferator-activated receptor (PPARg), reducing adipogenesis, insulin signaling, and glucose transport [24], coupled with antioxidant, anti-inflammatory, and antidepressant effects [25,26,27]. *S. elegans* caffeic and ferulic acids retarded weight gain in obese mice and reduced body weight gain, hyperglycemia, dyslipidemia, and hepatic steatosis in C57/BL6 MetS mice [28]. Furthermore, luteolin found in SeFAc decreased adipogenesis and insulin resistance, effects also exerted by this compound in ovariectomized mice fed with HFD [29].

Ethyl α-d-glucopyranoside, a compound present in SeF3, is another molecule that contributes to the activity against MetS exerted by S. elegans, specifically as an antihypertensive due to the diuretic effect it induces. This glycoside is one of the main compounds of the traditional Japanese alcoholic beverage denominated “sake” [30]. Additionally, it has been reported that this substance did not alter the standard blood glucose or insulin levels [31,32].

SeF3 contains phenol, 4-(3-hydroxy-1-propenyl)-2-methoxy (coniferyl alcohol), previously described in the species *Mussaenda frondosa* Linn. and *Feronia elephantum* Correa (Rubiaceae), widely employed for hypertension management due to its significant diuretic effect [33,34]. In addition, monolignin is included among the dietary fibers of cereals [35]. SeF3 was able to effectively counteract dyslipidemia, one of the main MetS features, due to sterol content (Stigmastan-3,5-dien; Stigmasta-5,22-dien-3-ol, acetate, (3β)-; Ergost-5-en-3-ol, acetate, (3β, 24*R*)-), because these compounds reduce the incorporation of dietary and biliary cholesterol into circulating micelles, which leads to a decrease in the synthesis and absorption of cholesterol, increases LDL receptor activity, which in turn reduces the serum concentration of LDL-cholesterol [36].

Moreover, the antihyperglycemic effect caused by SeF3 sterols has already been reported in diabetic mice [37,38], as well as the improvement of glucose tolerance in obese Zucker rats [39], which counteracts MetS. The antidepressant effect caused by these sterols detected in SeF3 has also been previously described. β-sitosterol and its derivatives have an antidepressant effect on FST and tail suspension tests in mice [25]. β-sitosterol (10, 21, 30 mg/kg) in male ICR mice decreased the immobility time of these animals in FST [40]. In addition, mice exposed to HFD increased their corticosterone levels, which led to the depressive behavior observed in FST [41,42]. Furthermore, the elevated level of this substance led to hyperglycemia and decreased insulin sensitivity, typical alterations in MS. Therefore, given the HFD-induced high corticosterone levels, this diet could be considered a stressor for mice [43]. The same effect was observed with FST exposure [44]. Therefore, the activities exerted by SeF3 in mice exposed to HFD may counteract MetS-related alterations and their associated depression.

SeF3 fraction contains 30% glycerol, considered an intermediate in lipid metabolism and stored as a precursor in triglyceride synthesis, mainly in adipose tissue. For example, during fatty food intake containing approximately 90% triglycerides, more than 30% of them are completely hydrolyzed to form free fatty acids and glycerol, which is released to the liver through aquaporins (AQP7 in the adipose tissue, and AQP9 in the liver), to be transformed into glycerol-3-phosphate (G3P). This compound is used for lipogenesis during fasting [45]. A cross-sectional and longitudinal study in a Finnish male population, it was shown showed that glycerol and monounsaturated fatty acids serum levels increase, which could serve as predictors of hypoglycemic exacerbation and the development of type 2 diabetes [46]. Glycerol also plays a vital role in liver metabolism since it incorporates glutathione, the primary antioxidant agent, and the liver is the main source of this molecule. The glycolytic pathway of glycerol leads to the formation of glycine by 3-phosphoglycerate. It can also be metabolized to pyruvate and enter the cyclic tricarboxylic acid cycle to form glutamate. Therefore, glycerol contributes to the *de novo* formation of glutathione through the amino acids. These recent data provide a therapeutic approach to this alcohol as a hepatoprotector by contributing to the increase of glutathione as an antioxidant [47].

Different homovanillic acid derivatives were measured for their inhibition of fatty acid uptake, one of them being methyl-2-(4-hydroxy-3-methoxy-3-methoxy-phenyl) acetate (methyl homovanillate, identified in the SeF3 fraction); there was no significant difference. The authors point out that the fatty acid ramified side chain, regardless of length, is a key element in enhancing fatty acid uptake in Caco-2 enterocytes [48]. Therefore, future studies must determine whether this compound constitutes an active principle of the SeF3 fraction.

MetS and depression resulting from the hypercaloric diet lead to inflammation and tissue damage due to macrophage recruitment and activation, with subsequent local and systemic release of proinflammatory cytokines. Increased levels of IL-1β, IL-6, and TNF-α have been observed in adipose tissue and serum of obese patients, which is associated with insulin resistance in humans and obese mice exposed to HFD. Increased expression of proinflammatory cytokines has been described in the hippocampus and hypothalamus, leading to macrophage infiltration into the Central Nervous system (CNS) [44,45]. In the spleen, the major lymphoid organ responsible for systemic immune response, TNF-α and IL-6 expression in this organ decreased significantly with obesity [49].

Likewise, a considerable decrease in the anti-inflammatory cytokine IL-10, which is responsible for inhibiting the synthesis of the above-mentioned proinflammatory cytokines, was also observed [50]. These inflammatory molecules, along with IL-1β, enhanced and promoted multisystemic inflammation in the hypothalamus of HFD-fed obese mice [51].

In white adipose tissue, reduced IL-10 led to chronic inflammation, contributing to insulin resistance. Splenectomy has been shown to exacerbate the inflammatory status of adipose tissue, kidneys, pancreas, and liver [52,53]. In chronic renal failure related to obesity, TNF-α, IL-1β, and IL-6 genetic overexpression was associated with chronic inflammation in adipose tissue. Also, it has been found that IL-6, derived from adipose tissue, exerts its effect on distant organs, including kidneys, promoting the development of oxidative stress and endothelial damage in obese mice [54,55]. Therefore, *S. elegans* may promote mitigation of cellular inflammation, mainly through increasing IL-10 concentration in the spleen, without modifying other proinflammatory molecules of the immune response. These data suggest that the active fractions regulate the inflammatory process and reduce obesity-associated renal damage.

It is necessary to continue the pharmacological study of this medicinal species, focusing on trying to elucidate the mechanism of action by which *S. elegans* decreases the damage caused by MetS and depression, giving continuity to the signaling process involved, which can be explored through in vitro techniques. It is also necessary to carry out the exhaustive separation of the plant for the isolation and structural elucidation of the secondary metabolites responsible for the activity against MetS. Additionally, pharmacokinetic and toxicological studies should be carried out to design a phytomedicine useful in the clinic.

## 4. Materials and Methods

### 4.1. Obtaining Plant Material, Hydroalcoholic Extract (SeHA) and Fractions SeF1, SeF2, and SeF3

*S. elegans* specimens collected in Ozumba, State of Mexico, Mexico (2340 m above sea level, masl) were identified by Margarita Avilés and Macrina Fuentes, and the INAH herbarium number M-2056 was assigned the specimens. The dry aerial parts (360 g) and ground (Pulvex) were macerated in ethanol: water 60:40 for 24 h.

The extract (SeHA) was dissolved in water (1.5 L), and ethyl acetate (2.5 L) was added, then they were concentrated in a rotary evaporator (Laborota 4000, Heidolph Instruments GmbH & Co. KG, Scwabach, Bavaria, Germany,) to obtain an aqueous fraction (SeFAq) and an organic fraction (SeFAc), obtaining 405 and 9.58 g, respectively. The SeFAc fraction (6 g) was absorbed on normal phase silica gel (15 g, 70–230; Merck KGaA, Darmstadt, Germany) and was fractionated by chromatography on a glass column (10 × 60 cm) packed with silica gel. The elution system had a gradient with hexane and a 10% polarity increase with ethyl acetate and washed with methanol, collecting volumes of 50 mL to give 34 fractions. TLC monitored the separation, and they were pooled according to the similarity in chemical content into three fractions: SeF1 (1–8, 450 mg), SeF2 (9–22, 638 mg), and SeF3 (23–33, 745 mg).

### 4.2. HPLC Analysis of the Extract and Fraction

HPLC analysis of SeHA and SeFAc was performed in Waters 2695 equipment, coupled with a photodiode detector (Waters model 2996), supported by Empower 3 software (Version 3, Waters Corporation, Milford, MA, USA). The stationary phase was the Chromolith Performance RP-18e column (4.6 × 100 mm, Merck, Germany). Gradient elution was carried out with 0.5% trifluoroacetic acid (solvent A) and acetonitrile (solvent B). The gradient was: 0–1 min, 0% B; 2–4 min, 10% B; 5–7 min, 20% B; 8–14 min, 30% B; 15–18 min, 40% B; 19–22 min, 80% B; 23–26 min, 100% B, and 27–28 min, 0% B. The flow rate was 1.0 mL/min, and the injection volume was 10 μL of the sample in methanol.

### 4.3. SeF3 GC-MS Analysis

SeF3 was analyzed by gas-mass chromatography (GC-MS) (equipment brand) coupled to a quadrupole mass detector in electron impact mode at 70 eV on an HP-5 ms capillary column (Agilent, J&W Ultra Low-Bleed (Q) GC/MS columns, Santa Clara, CA, USA) (25 µm in length, 0.2 mm id, 0.3 µm in film thickness); the programmed temperature programmed was 40 °C for 2 min, subsequently, the temperature is increased from 40 °C to 260 °C in 10 min and remained at 260 °C for 20 min. The mass detector was programmed with the interface temperature at 200 °C and with a mass acquisition range of 20–550. The injector and detector temperatures were 250 and 280 °C, respectively. The volume of the sample that was injected was 1 µL at a concentration of 3 mg/mL. The mobile phase was helium gas at a 1 mL/min flow rate. The compounds were identified to compare the mass spectra with those of the National Institute of Standards and Technology (NIST, 1.7 Library).

### 4.4. Treatments

The treatment was SeHA (100 mg/kg), SeFAc (50 mg/kg), and SeF3 (10 mg/kg); different drugs were used, including the following: Imipramine (IMI, antidepressant at 10 mg/kg, 98% purity, Sigma-Aldrich, St. Louis, MO, USA), Telmisartan (TEL, antihypertensive to 10 mg/kg, 98% purity, Sigma-Aldrich), Glibenclamide-Metformin (G/M, an antidiabetic, 1/100 mg/kg, Medimart, Montreal, QC, Canada) and Pravastatin (PRA, an antidyslipidemic, 2 mg/kg, Medimart). A Tween 20 solution (Tw 1%, Merck) was employed for the negative control group.

### 4.5. Animals

Newly weaned male ICR mice were obtained from Envigo S.A. de C.V. (Mexico City, Mexico). They were maintained for 3 weeks with a 12 h:12 h light-darkness cycle and free access to water and food (standard diet, Std, pellets from Envigo Rodent Lab Diet, composed of 4% lipids, 22% proteins, and 42.7% carbohydrates). After the mice were organized into groups (n = 12), all animals, except for the healthy group of mice with S.D., received a high-fat diet (HFD, composed of 20% lipids, 20% proteins, and 26.4% carbohydrates) for 12 weeks.

All tests followed the Federal Regulations for Animal Experimentation and Care (Ministry of Agriculture, NOM-062-ZOO-1999, Mexico City, Mexico). The Research and Ethical Committee of the Mexican Institute of Social Security approved the experimental protocol (R-2019-1702-041).

### 4.6. Experimental Design

(1) Healthy mice only consumed the standard diet—StD;
(2) Obese group, with only a high-fat diet [VEH];
(3) Antihypertensive control group, with a high-fat diet and Telmisartan [TEL, 10 mg/kg];
(4) Antidepressive control group, with high-fat diet and Imipramine [IMI, 10 mg/kg];
(5) Antidiabetic control group antidiabetic, with a high-fat diet and Glibenclamide/Metformine [G/M, 1/100 mg/kg];
(6) Antihypercholesteremic control group, with a high-fat diet and Pravastatin [PRV, 2.0 mg/kg];
(7) Experimental group, with a high-fat diet and SeHA 100 mg/kg;
(8) Experimental group, with a high-fat diet and SeFAc 50 mg/kg;
(9) Experimental group, with a high-fat diet and SeF3 10 mg/kg.

During the experiment, weight gain was recorded three times each week to establish the growth curve and the animal’s food consumption. Blood pressure was measured throughout the experimentation in weeks 8 and 12. At the end of week 12, the depression-like behavior was measured in the forced swimming test (FST). After animals were deeply anesthetized intraperitoneally (i.p.) with pentobarbital sodium at 60 mg/kg, blood samples were quickly obtained from the retroorbital sinus and stored under 4 °C until use. The serum was obtained by centrifugation (3500 rpm for 7 min). Then, the samples were stored individually at −20 °C to obtain various determinations (glucose, cholesterol, triglycerides, and corticosterone). The brain, spleen, kidneys, and visceral fat were dissected for inflammation by quantifying cytokines such as TNF-α, IL-1β, IL-6, and IL-10 by the ELISA method. The following parameters were also measured: the adiposity index and the insulin resistance (as the area under the curve [AUC]).

### 4.7. Forced Swimming Test (FST)

FST is used for assessing antidepressant activity [16,18]. The apparatus consisted of a transparent Plexiglas cylinder (24 cm × 12 cm in diameter) filled to a depth of 15 cm with water (25 ± 2 °C). The test consists of a pre-test session of 15 min and, 24 h later, a test session of 5 min. In this design, since the HFD generates the depression, a modification was carried out on the test, skipping the pre-test session and conducting the test session for only 7 min. During this, the immobility time was registered, considered to be when the mouse made no further attempts to escape.

### 4.8. Measuring Blood Pressure

The mice exposed to different treatments during this essay were anesthetized (pentobarbital sodium, 55 mg/kg i.p. Pfizer, Ciudad de México, Mexico) and placed in a LE 5002 LETICA Storage Pressure Meter (Biopac System MP150, Goleta, CA, USA). Ten measurements were recorded for each mouse, and an average was obtained to report the systolic and diastolic pressure.

### 4.9. Determination of Body Density

Under surgical anesthesia (pentobarbital sodium, 55 mg/kg i.p., Pfizer, Mexico), the mice were immersed in a graduated volumetric device with 100 mL of water, keeping the nose of the rodents out of the water. The volume of displaced water is measured and recorded; the ratio between weight and displaced volume is calculated, and this index corresponds to body density.

### 4.10. Determination of Fasting Glucose

To determine the effect of the glucose concentration of the animals to different treatments, previously fasted for 12 h, a blood sample (5 µL) was obtained from the tail vein, and the glucose was quantified (mg/dL) using a glucometer Bayer Contour^®^ TS glucometer (Bayer HealthCare LLC Mishawaka, IN, USA).

### 4.11. Insulin Tolerance Curve

The insulin tolerance curve (ITC) was executed at the end of the treatments. In mice previously fasted for 12 h, 5 µL of blood was obtained from the tail vein; the glucose was quantified (mg/dL) utilizing a Bayer Contour TS flucometer. Afterward, a dose of 0.1 IU/kg of rapid insulin was applied (Humulin R U-500- Eli Lilly Corporate Center, Indianapolis, IN, USA) i.p. The glucose was quantified at 15, 30, 60, and 120 min after the application. The response curve was constructed with the data obtained, and the AUC was calculated.

### 4.12. Plasmatic Cholesterol and Triglyceride

The plasmatic concentration of cholesterol and triglycerides was determined in plasma by enzymatic colorimetric assay using the Wiener Lab (Rosario, Santa Fe, Argentina) Color Colestat AA and TG GPO/PAP AA, following the recommendations of the supplier. The plates were read in an ELISA reader (Probiotek, San Nicolás de los Garza, Mexico) model ELx800, and sample absorbance at 492 nm was recorded.

### 4.13. Corticosterone Determination

The plasmatic concentration of corticosterone was determined by means of an ELISA assay employing the ALPCO (Salem, NH, USA) corticosterone ELISA kit, following the supplier’s recommendations. The plate was read in a model ELx800 ELISA reader (Probiotek), and the sample absorbance at 492 nm was recorded.

### 4.14. Quantification of Quantification of Cytokines in Kidneys, Adipose Tissue, Brain, and Spleen

The kidneys and adipose tissue of the mice were transferred to 15 mL tubes, which were placed on dry ice and suspended in phenyl methyl sulfonyl fluoride (PMSF; Sigma-Aldrich) 0.1 mM in PBS (1 mL/200 mg of tissue). Samples were homogenized (Potter-Elvehjem homogenizer, Thomas Scientific, Swedesboro, NJ, USA) and then centrifuged for 10 min at 14,000 rpm at 4 °C. The supernatants were transferred into 1.5 mL Eppendorf tubes and were ready for analysis. IL-1β, IL-6, IL-10, and TNFα were measured using ELISA BD OptEIA™ (San Jose, CA, USA), according to the manufacturer’s instructions. Concentrations for each cytokine were calculated from calibration curves using individual recombinant proteins as standards, also according to the manufacturer’s instructions. Density (OD) readings were performed at 30 and 60 min of incubation at 450 nm. The concentration of pro-inflammatory cytokines (IL-1β, IL-6, and TNFα) was divided by the value of the anti-inflammatory cytokine (IL-10). Finally, the resulting index was compared with the baseline value.

### 4.15. Statistical Analysis

Statistical analysis was performed with an SPSS ver. 17.0 software program (SPSS Inc. Released 2008. SPSS Statistics for Windows, Version 17.0. Chicago: SPSS Inc., Chicago, IL, USA), and the results were expressed as mean-standard deviation (SD). Data were analyzed by Analysis of variance (ANOVA) followed by the *post hoc* Bonferroni test (* *p* < 0.05, compared to the VEH group, ^&^ *p* ≤ 0.05 compared to the StD group).

## 5. Conclusions

Dyslipidemia is a central component of MetS, which causes organic damage to various target organs. Chronic administration of a hypercaloric diet in mice first induced a considerable increase in adipose tissue and elevated serum lipid levels. This condition triggers a low-grade systemic proinflammatory environment, damaging various organs and tissues, including the kidneys, spleen, brain, and adipose tissue. However, it should be emphasized that the endothelium, an organ throughout the body, is irreversibly damaged, leading to a broad-spectrum deleterious condition known as endothelial dysfunction, which explains why dyslipidemia, as a component of MetS, is a risk factor for diseases such as myocardial infarction, stroke, and kidney failure. Based on the above, it can be considered that the treatments using the extract of *S. elegans*, with rich content of glycosides, sterols, flavonoids, and phenolic acids, were able to mitigate the damage process by acting in principle as an antidyslipidemic, antiobesity, antihyperglycemic and capable of reducing insulin resistance, and as a consequence diminished the alterations derived from endothelial dysfunction, thereby reducing low-grade systemic inflammation. Protecting the vasculature and damage in different target organs. In addition, it modulated serum levels of corticosterone, a measurement factor of the overactivation of the HPA axis, and therefore acts as an antidepressant.

## Figures and Tables

**Table 1:** Chemical composition of SeF3 from *Salvia elegans* determined by GS-MS.

Compound Name	Molecular Weight (a.m.u.)	RT (min)	Concentration (% of Total)
Glycerol (Glycerine)	92.09	7.29	33.4
Ethyl α-d-glucopyranoside	208.21	15.826	21.36
Methyl homovanillate	196.07	16.83	2.97
Coniferol	180.20	16.982	5.96
Campesterol acetate	382	31.33	6.39
Stigmasterol acetate	454.7	31.75	4.20

**Table 2:** Effect of drugs and *S. elegans* on different parameters of MetS-induced with HFD.

Treatments	Weigh Gain (Velocity g/Days)	Blood Pressure (mmHg)				Other Parameters Measured at Week 12				
	Week 12	Week 8		Week 12		Glucose (mg/dL)	Triglycerides (mg/dL)	Cholesterol (mg/dL)	Insulin Tolerance Curve (AUC)	Adiposity Index (%)
		SBP	DBP	SBP	DBP					
StD	0.196 ± 0.01 *	99 ± 17 *	59 ± 7 *	93 ± 14 *	60 ± 7.3 *	80 ± 15 *	29 ± 11 *	156 ± 34 *	5383 ± 1695 *	0.4 ± 0.2 *
VEH	0.240 ± 0.017 ^&^	147 ± 17 ^&^	80 ± 15 ^&^	146 ± 13 ^&^	78 ± 8 ^&^	115 ± 9 ^&^	63 ± 12 ^&^	248 ± 14.0 ^&^	16,633 ± 2094 ^&^	1.5 ± 0.6 ^&^
IMI	0.161 ± 0.031 *	140 ± 37 ^&^	79 ± 7 ^&^	139 ± 29 ^&^	70 ± 8.6	106 ± 20 ^&^	36 ± 15 *	233 ± 57 ^&^	7835 ± 4517 * ^&^	1.3 ± 0.26 ^&^
G/M	0.155 ± 0.024 *	121 ± 36 ^&^	67 ± 7 *	101 ± 19 *	63 ± 11	85.6 ± 19.5 *	26 ± 12 *	185 ± 57	8388 ± 1047 * ^&^	1.31 ± 0.3 ^&^
PRV	0.165 ± 0.02 *	143 ± 33 ^&^	65 ± 9 *	123 ± 13 ^&^	67 ± 11	97.1 ± 13.4 *	21 ± 13 *	165 ± 52 *	13,131 ± 1363 * ^&^	0.68 ± 0.12 *
TEL	0.189 ± 0.03 *	99 ± 18 *	60 ± 6 *	100 ± 16 *	62 ± 4.5 *	149 ± 22 * ^&^	27 ± 13 *	174 ± 50 *	3423 ± 435 * ^&^	2.1 ± 0.58 ^&^
SeHA 100	0.215 ± 0.032 ^&^	107 ± 18 *	60 ± 5.8 *	105 ± 19.8 *	60 ± 8.0 *	96 ± 17.7 ^&^	50.75 ± 9.6 ^&^	160 ± 43.7 *	11,709 ± 1639 * ^&^	1.67 ± 0.25 ^&^
SeFAc 50	0.149 ± 0.020 *	106 ± 22.8 *	59 ± 6.5 *	110 ± 22.1 * ^&^	60 ± 3.4 *	96 ± 8.8 ^&^	44.34 ± 9.13 *	137.02 ± 55 *	11,729 ± 2764 *	1.58 ± 0.28 ^&^
SeF3 10	0.168 ± 0.064 *	103 ± 19.6 *	61 ± 4.7 *	102 ± 16.0 *	62 ± 7.9 *	84 ± 12.7 *	7.72 ± 4.8 * ^&^	188.87 ± 8.9 *	8655.5 ± 2880 *	0.77 ± 0.13 *

Note: With to exception StD group, all animals consumed the HFD diet daily. ANOVA post-test Bonferroni (*, ^&^ *p* ≤ 0.05; *n* = 10). StD = standard diet; VEH = animals that received only HFD; IMI = Imipramine; G/M = glibenclamide plus metformin; PRV = Pravastatin; TEL = Telmisartan; SeHA = hydroalcoholic extract; SeFAc = acetate of ethyl fraction; SeF3 = sub-fraction from SeFAc.

**Table 3:** Effect of *S. elegans* on left and right kidney cytokine levels in HFD mice.

Organ	Treatment (mg/kg)	TNF-α	IL-1β	IL-6	IL-10
		(pg/mg prot)			
Left kidney	StD	150.13 ± 38.02 *	22,648.7 ± 2092.5 *	491.18 ± 31.34 *	1682.7 ± 26.5 *
	VEH	2502.07 ± 15.73	57,756.3 ± 625.2	1130.42 ± 72.1	4150.8 ± 743
	IMI 1.0	328.35 ± 10.55 *	13,679.9 ± 1536.8 *	561.4 ± 297.0 *	1524.7 ± 87.1 *
	G/M (1/100)	550.79 ± 35.08 *	28,849.6 ± 4688.2 *	706.7 ± 101.5 *	2346.2 ± 233.9 *
	PRAV 2.0	571.03 ± 89.58 *	21,883.3 ± 18.8 *	547.2 ± 125.5 *	2859.1 ± 232.3 *
	TEL 10	213.80 ± 83 *	18,914.03 ± 2950 *	435.9 ± 92.2 *	3134.1 ± 358.9
	SeHA 100	1977.9 ± 457.4	36,147.7 ± 2629.9 *	1046.16 ± 520.1	5398.7 ± 208.3 *
	SeFAc 50	182.52 ± 76.5 *	15,396.0 ± 668.1 *	2267.1 ± 426.0 *	2267.1 ± 426.0 *
	SeF3 10	172.62 ± 37.1 *	387.14 ± 60.9 *	387.1 ± 60.9 *	1504.4 ± 6.4 *
Right kidney	StD	59.86 ± 9.29 *	21,618.1 ± 2908.5 *	799.24 ± 114.9 *	2232.6 ± 129.1 *
	VEH	107.06 ± 3.02	39,068.8 ± 3099.5	1261.90 ± 78.40	3899.7 ± 113.8
	IMI 1.0	150.82 ± 21.14 *	10,978.5 ± 986.4 *	463.61 ± 163.9 *	2432.08 ± 157.1 *
	G/M (1/100)	642.32 ± 79.5 *	24,799.4 ± 3321.4 *	803.8 ± 94.5 *	3250.62 ± 682.2
	PRAV 2.0	467.69 ± 120.0 *	34,178.6 ± 1866.2	777.5 ± 50.8 *	4853.4 ± 365.1 *
	TEL 10	222.14 ± 7.56 *	17,041.9 ± 1440 *	440.60 ± 61.62 *	2407.19 ± 88.6 *
	SeHA 100	206.66 ± 77.5 *	32,445 ± 2966.0 *	1286.9 ± 43.9	3668.1 ± 146.0
	SeFAc 50	393.9 ± 17.7 *	31,371.8 ± 942 *	1184.39 ± 195.2	3505.5 ± 198.3 *
	SeF3 10	74.10 ± 24.5 *	24,913.0 ± 4098.3 *	1069.7 ± 14.9 *	3442.4 ± 116.7 *

Treatments deriving from *S. elegans* (SeHA = hydroalcoholic extract; SeFAc = acetate of ethyl fraction; SeF3 = sub-fraction from SeFAc; IMI = Imipramine; G/M = Glibenclamide/Metformin; PRAV = Pravastatin; TEL = Telmisartan). ANOVA post-test Bonferroni (* *p* ≤ 0.05, in comparison with the VEH group; *n* = 10).

**Table 4:** Effect of *S. elegans* on white adipose tissue cytokine levels in HFD mice.

Organ	Treatment (mg/kg)	TNF-α	IL-1β	IL-6	IL-10
		(pg/mg prot)			
White fat tissue	StD	282.98 ± 18.04 *	21,283.3 ± 1084.1 *	869.13 ± 47.84 *	468.8 ± 33.8 *
	VEH	2641.4 ± 556.5	85,245.4 ± 4433	1522.5 ± 361.5	1578.7 ± 37.27
	IMI 1.0	1419.68 ± 20.42 *	53,589 ± 6303.2 *	707.1 ± 107.4 *	2480.9 ± 402.5 *
	G/M (1/100)	2400.8 ± 222.2	36,109.3 ± 4369.6 *	1753.2 ± 131.7	1265.51 ± 214.5
	PRAV 2.0	1448.5 ± 408.05 *	68,698.3 ± 20,143	1772.19 ± 147.2	1831.9 ± 391.5
	TEL 10	192.33 ± 12.17 *	34,745 ± 5161 *	834.1 ± 9.0 *	2203.7 ± 140 *
	SeHA 100	1087.9 ± 89.36 *	39,222.7 ± 4145.6 *	1494.41 ± 104.2	1333.7 ± 43.2
	SeFAc 50	1308.2 ± 45.34 *	54,865 ± 1486.5 *	692.42 ± 192.6 *	344.8 ± 49.3 *
	SeF3 10	953.05 ± 22.7 *	36,919.7 ± 9986 *	674.2 ± 75.9 *	1880.2 ± 623.5

Treatments from *S. elegans* (SeHA = hydroalcoholic extract; SeFAc = acetate of ethyl fraction; SeF3 = sub-fraction from SeFAc; IMI = Imipramine; G/M = Glibenclamide/Metformin; PRAV = Pravastatin; TEL = Telmisartan). ANOVA post-test Bonferroni (* *p* ≤ 0.05, in comparison with VEH group, *n* = 10).

**Table 5:** Effect of *S. elegans* on brain cytokine levels in HFD mice.

Organ	Treatment (mg/kg)	TNF-α	IL-1β	IL-6	IL-10
		(pg/mg prot)			
Brain	StD	603.15 ± 38.07 *	1779.7 ± 22.9 *	274.3 ± 59.3 *	940.1 ± 128.5 *
	VEH	884.16 ± 27.87	2455.3 ± 332.1	494.51 ± 40.10	430.6 ± 167
	IMI 1.0	211.83 ± 19.83 *	3576.2 ± 1257	245.0 ± 25.7 *	871.7 ± 68.0 *
	G/M (1/100)	766.71 ± 125.46	3619.0 ± 215.6 *	214.7 ± 6.3 *	397.26 ± 79.4
	PRAV 2.0	344.09 ± 75.1 *	1824.8 ± 213.9 *	222.41 ± 5.63 *	456.5 ± 158.4
	TEL 10	63.03 ± 7.83 *	4531.7 ± 137.8 *	273.4 ± 40 *	930.9 ± 35.2 *
	SeHA 100	763.75 ± 58.45 *	2106.7 ± 848	238.45 ± 21.17 *	895.8 ± 151.4 *
	SeFAc 50	1284.69 ± 130.1 *	3033.2 ± 509.2	280.2 ± 45 *	929.8 ± 118.9 *
	SeF3 10	1032.01 ± 239.02	3204.0 ± 506.0	305.4 ± 67.4 *	609.1 ± 55.9 *

Treatments from *S. elegans* (SeHA = hydroalcoholic extract; SeFAc = acetate of ethyl fraction; SeF3 = sub-fraction from SeFAc; IMI = Imipramine; G/M = Glibenclamide/Metformin; PRAV = Pravastatine; TEL = Telmisartan). ANOVA post-test Bonferroni (* *p* ≤ 0.05, in comparison with the VEH group; *n* = 10).

**Table 6:** Effect of *S. elegans* on spleen cytokine levels in HFD mice.

Organ	Treatment (mg/kg)	TNF-α	IL-1β	IL-6	IL-10
		(pg/mg prot)			
Spleen	StD	731.70 ± 41.09 *	24,613.9 ± 826.6 *	796.80 ± 30.34 *	1499.4 ± 3.3 *
	VEH	299.75 ± 64.9	12,326.3 ± 581.2	215.51 ± 12.59	201.60 ± 48.8
	IMI 1.0	123.29 ± 7.4 *	7072.7 ± 1246 *	337.53 ± 35.18 *	976.2 ± 22.9 *
	G/M (1/100)	1265.98 ± 90.4 *	14,151.6 ± 686.4 *	444.12 ± 9.41 *	1510.5 ± 40.8 *
	PRAV 2.0	365.5 ± 79	11,152.3 ± 1968	424.35 ± 20.88 *	1270.3 ± 195.6 *
	TEL 10	1920 ± 218.2 *	6582.1 ± 145.2 *	492.62 ± 21.94 *	991.4 ± 65.2 *
	SeHA 100	331.5 ± 28.4	10,250.3 ± 2215.0	205.6 ± 31.3	411.1 ± 2.05 *
	SeFAc 50	242.1 ± 1.3	11,127.4 ± 1808	224.69 ± 12.1	519.6 ± 96.0 *
	SeF3 10	212.89 ± 56.7	6660.4 ± 607.5 *	221.31 ± 93.3	636.8 ± 37.0 *

Treatments from *S. elegans* (SeHA = hydroalcoholic extract; SeFAc = acetate of ethyl fraction; SeF3 = sub-fraction from SeFAc; IMI = Imipramine; G/M = Glibenclamide/Metformin; PRAV = Pravastatine; TEL = telmisartan). ANOVA post-test Bonferroni (* *p* ≤ 0.05, in comparison with the VEH group; *n* = 10).

## Data Availability

Data are contained within the article.
